# The relationship between the structural characteristics of lactobacilli-EPS and its ability to induce apoptosis in colon cancer cells *in vitro*

**DOI:** 10.1038/s41598-019-44753-8

**Published:** 2019-06-04

**Authors:** Ummugulsum Tukenmez, Busra Aktas, Belma Aslim, Serkan Yavuz

**Affiliations:** 1 Faculty of Science, Department of Biology, Gazi University, Ankara, Turkey; 2 Faculty of Arts and Science, Department of Molecular Biology and Genetics, Burdur Mehmet Akif Ersoy University, Burdur, Turkey; 3 Faculty of Science, Department of Chemistry, Gazi University, Ankara, Turkey

**Keywords:** Bacterial secretion, Industrial microbiology

## Abstract

Colon cancer is one of the most common cancer around the world. Exopolysaccharides (EPSs) produced by lactobacilli as potential prebiotics have been found to have an anti-tumor effect. In this study, lyophilized EPSs of four *Lactobacillus* spp. for their impact on apoptosis in colon cancer cells (HT-29) was evaluated using flow cytometry. The relationship between capability of a lactobacilli-EPS to induce apoptosis and their monosaccharide composition, molecular weight (MW), and linkage type was investigated by HPLC, SEC, and NMR, respectively. Changes in apoptotic-markers were examined by qPCR and Western Blotting. EPSs were capable of inhibiting proliferation in a time-dependent manner and induced apoptosis via increasing the expression of Bax, Caspase 3 and 9 while decreasing Bcl-2 and Survivin. All EPSs contained mannose, glucose, and N-acetylglucosamine with different relative proportions. Some contained arabinose or fructose. MW ranged from 10^2^–10^4^Da with two or three fractions. EPS of *L. delbrueckii* ssp. *bulgaricus* B3 having the highest amount of mannose and the lowest amount of glucose, showed the highest apoptosis induction. In conclusion, lactobacilli-EPSs inhibit cell proliferation in HT-29 via apoptosis. Results suggest that a relationship exists between the ability of EPS to induce apoptosis and its mannose and glucose composition.

## Introduction

Today, cancer is one of the most important health problem leading to death. Colorectal cancer is the second most common cause of cancer deaths in the world^1,2^. Patients with colorectal cancer have been treated with surgery, radiotherapy, or chemotherapy with toxic drugs such as 5-fluorouracil and oxaliplatin. Most of the anti-cancer drugs used in chemotherapy cause immunotoxicity and slow down the healing process^3^. Identification of anti-tumor therapy with low side effects is essential in cancer studies. In preventing colon cancer, therefore, diet style rich in fibers, fermented foods such as probiotics have been suggested^3^.

Gut microbiota has found to be related to the risk of colorectal cancer^4^. A well-balanced bidirectional relationship between gut microbiota and host immune system exists^5^. Disruption of this balance with dysbiosis, the perturbation of the healthy/normal gut microbiota, can result in a wide range of disorders or diseases such as metabolic disorders and inflammatory bowel diseases^6,7^. A study on patients with colorectal cancer showed that the abundance of *Fusobacteria* was enriched in the colon tissue with tumor^8^. Zackular *et al*. reported that abnormal microbiota in mice is associated with inflammation and tumor in colon^9^. Another study showed that patients with colon cancer had lower counts of total *Bifidobacterium* in the colon microbiota^4^. It was assumed that targeted alterations in the gut microbiota could be used to prevent or treat colorectal cancer in the future^4,10^.

Probiotics are one of the strategies that could be used to alter the microbial composition of the gut. A diverse set of health benefits have been ascribed to probiotics including: immunomodulation, improved ability to tolerate lactose; reduction in gastrointestinal pathogens; and reduction in colorectal cancer^11–13^. Probiotics come from a variety of genera, including *Lactobacillus, Bifidobacterium, Propionibacterium, Escherichia*, and *Saccharomyces*; however, *Lactobacillus* are the most common genera used as probiotics^14^. Studies have demonstrated that bacterial secondary products can also alter the gut environment and affect cancer development^15,16^. In a study evaluating the impact of cell fractions and exopolysaccharides (EPSs) from *Lactobacillus* on colon cancer cells, Liu *et al*. reported that EPS has the highest cytotoxic effect among the tested fractions and reduced proliferation of colon cancer cells^17^. Another study investigating the effect of lactobacilli EPS on cervical tumor cells demonstrated that lactobacilli EPS induced apoptosis in tumor cells and showed an anti-proliferative effect^15^. Microbial EPSs are primary or secondary metabolites produced by microorganisms and they have been used as prebiotics, which defined as the specific fermented ingredient resulting in changes in the gastrointestinal microbiota and provide health benefit^18,19^. There is a great diversity among EPS produced by lactic acid bacteria (LAB)^20^. LAB EPSs can be divided into homopolysaccharides (HoPSs) consisting one type of monosaccharide, and heteropolysaccharides (HePSs) consisting a backbone of repeating units that are composed of two or more types of monosaccharides^21,22^. LAB mostly produces HePSs which consist of different sugars such as pentose (D-arabinose, D-ribose, D-xylose), hexose (D-glucose, D-galactose, D-mannose), or uronic acids (D- glucuronic acid, D-galacturonic acid). They mostly consist different types of linkages and branches such as α-(1,2) or α-(1,6) linkages which are rigid, β-(1,4) or β-(1,3) which are less rigid^23,24^. Studies reported that the composition and the structure of EPSs tend to be strain dependent^25,26^. Moreover, it has been shown that structural and compositional diversity among EPSs might be responsible for the variation in their health benefits^20,27^. Li *et al*. purified three fractions of EPS isolated from *L. helveticus* MB2-1 and evaluated their structure and antioxidant activities *in vitro*^28^. They reported that although the molecular weights of EPSs were similar, their sugar compositions and antioxidant effect were different as well as their anti-cancer impact on colon cancer cells^28,29^. Additionally, anti-cancer activity of polysaccharides can be affected by other physicochemical properties, such as presence of β-type glycosidic linkages, uronic acid, sulfate groups, and glucose increasing anti-cancer activity^30–33^. Therefore, the determination of chemical composition and structure of EPS must be taken into consideration when predicting potential applications of EPSs^30^.

Although lactobacilli EPSs have been reported to have cytotoxic effect on various cancer cell lines, the mechanism of action and the impact of its structure on cytotoxic effect have not been understood yet. In this study, we investigated the EPS produced by various *Lactobacillus* spp. and their impact on proliferation and apoptosis in colon cancer cells. We performed a chemical and structural characterization of lactobacilli EPSs including molecular weight, monosaccharide composition, and linkage type. Additionally, we evaluated their structural, characteristic effects on apoptosis.

## Materials and Methods

### Bacterial strains

A total of four previously described *Lactobacillus* spp. isolated from healthy infant feces (*L. plantarum* GD2, *L. rhamnosus* E9, *L. brevis* LB63) and yogurt (*L. delbrueckii* ssp. *bulgaricus* B3) were used in this study^34,35^. Bacterial species were confirmed by 16S rRNA sequence analysis using universal primers (Uni27F, 5′ AGAGTTTGATCCTGGCTCAG 3′ and Uni1492R, 5′ GGTTACCTTGTTACGACTT 3′). Lactobacilli stock cultures were maintained at −30 °C in MRS broth (Oxoid, Istanbul, Turkey) with 10% (v/v) glycerol. Working cultures were prepared from frozen stocks by two sequential transfers in MRS broth and incubations were conducted aerobically at 37 °C for 18 h.

### Isolation and lyophilization of exopolysaccharide

The method of Frengova *et al*. was followed to isolate EPS^36^. The growth culture with an optical density of 0.6 at 600 nm (~8.5 log CFU/ml) was heated at 100 °C for 15 min. After cooling, the cell suspension was treated with 17% (v/v) of 85% trichloracetic acid solution and centrifuged at 15, 493 × *g* for 20 min to remove cells and proteins. The exopolysaccharide was precipitated using two volume of cold absolute ethanol followed by centrifugation at 15, 493 × *g* for 15 min. The resulting pellet containing EPS was suspended in deionized water. Total carbohydrate was measured at 490 nm by phenol-sulfuric acid method^37^ using glucose as standard. The EPSs isolated were stored at −80 °C until being lyophilized in Christ Alpha 2–4 freeze dryer (Marin Christ Co. FL, USA). The freeze-dried EPS powder was stored at 4 °C^38^.

### Physico-chemical characterization of the EPS produced by *Lactobacillus* strains

#### Monosaccharide composition

The method of Ledezma *et al*. was followed to hydrolyze EPSs isolated from *Lactobacillus* spp.^39^. Briefly, EPSs (10 mg/ml) were incubated with 1 M H_2_SO_4_ for 3 hours at 90 °C and then neutralized with 1 M NaOH to pH 7. After the complete hydrolysis, monosaccharide composition of EPS isolated from *Lactobacillus* spp. was quantified by high pressure liquid chromatography (HPLC) using an AGILENT 1260 system equipped with a refractive index detector at the Middle East Technical University, Central Laboratory. The separation (25 µl volume of injection) was carried out in the Metacarb 67 C columns (300 mm × 6.5 mm) maintained at 90 °C. For N-acetylglucosamine composition, the separation was carried out in the Metacarb 87 H (300 mm × 7.8 mm) column.

The mobile phase was water with a fixed flow rate of 0.5 ml/min and the separation was utilized for 30 min. Runs were performed at least in triplicate and the data was presented as mean ± SEM.

#### Molecular weight

This analysis was performed at the Bilkent University, National Nanotechnology Research Center (UNAM) following the protocol of Boymirzaev e*t al*.^40^. Molecular weight of EPSs was determined by Size Exclusion Chromatography (SEC) with an Agilent 1200 series system equipped with a PL aquagel-OH MIXED-H column and a refractive index detector. 10 µl of EPS at 0,05–0,2% (w/V) was injected and was eluted with 0.2–0.8 M NaNO_3_ at a flow rate of 0.6 ml/min. Polysaccharide (pullulan) was used as standard.

#### Structural analysis

Nuclear Magnetic Resonance Spectroscopy Analysis (NMR) was performed at Çankırı Karatekin University Research Center. NMR spectrum of the EPS isolated from *Lactobacillus* spp. (30 mg/500 µl) was recorded with 99.96% D_2_O as the solvent at 600 MHz (Agilent, 600 MHz, 14.1 Tesla Premium Compact NMR). Two-dimensional (2D) ^1^H–^1^H correlated spectroscopy (COSY), and nuclear overhauser effect spectroscopy (NOESY) measurements were used to assign signals and to determine the sequence of sugar residues. Spectra was referenced to internal trimethylsilylpropanoic acid^41^.

### Cell culture

HT-29, Human Colorectal Adenocarcinoma Cell Line, (ATCC® HTB-38™) was kindly provided by Prof. Hakan Akbulut (Ankara University, Medical Oncology). The cells were grown in Dulbecco’s modified Eagle’s medium **(**ThermoFisher Scientific**)** containing high glucose (4.5 g/l), sodium pyruvate (1 mM), and supplemented with 10% of heat-inactivated fetal bovine serum (ThermoFisher Scientific), penicillin/streptomycin (100 units/ml of penicillin and 100 μg/ml of streptomycin) (ThermoFisher Scientific), and L-glutamine (2 mM) (ThermoFisher Scientific). HT-29 cell culturing was carried out in 25 cm^2^ or 75 cm^2^ cell culture flasks at 37 °C in a humidified incubator with 5% CO_2_ atmosphere. The cell culture medium was changed every 48 h from the second day after seeding, and cells were harvested by 0.05% trypsin/EDTA (ThermoFisher Scientific) after reached 80–90% confluence.

### Anti-proliferation activity

Impact of EPSs from lactobacilli on HT-29 cell proliferation was evaluated using a WST-1 cell proliferation assay kit (Cayman Chemical Company, Ann Arbor, Michigan, USA). The lyophilized EPSs were dissolved in distilled water and filtered using a 0.2 µm syringe filter prior to analyses. HT-29 cells were seeded into a 96-well plate at a density of 1 × 10^4^ cells/well and treated with EPSs at a final concentration of 400 µg/ml followed by 24 h or 48 h incubation at 37 °C with 95% air and 5% CO_2_. After incubation, 10 μl of the WST-1 mixture was added to each well and the plates were incubated for 2 h at 37 °C with 95% air and 5% CO_2_. Formation of formazan was measured at 450 nm by a microplate reader (Epoch, Biotek, Winooski, VT, USA) and the absorbance was correlated with the cell number. The anti-proliferative effect was evaluated by comparing to viability of the treated samples with the untreated control (ultrapure water and DMEM mix without test sample for EPS). The percentage of viability was calculated as follows:

$$ \% {\rm{Viability}}=({\rm{Absorbance}}\,\mathrm{Sample}/\mathrm{Absorbance}\,{\rm{Control}})\,\times \,100$$

### Cell distribution by flow cytometry

Briefly, treated and untreated cells (control cells) were washed twice with PBS and harvested by scraping from 6 well plate using a cell scraper in PBS and collected by centrifugation (367 × *g*, 4 min). Flow cytometry analysis were performed according to kit manufacturer’s directions^42,43^. The cell pellets were resuspended into 1 ml of DMEM. Following that, 4 ml of Annexin V binding buffer was added (Cat. No: BB10X-50ml, Immunostep, Spain) and centrifuged (500 × *g*, 5 min). After centrifugation, the supernatant was aspirated and the cells were resuspended in 200 µl of 1X BB (BB10X diluted 1X with distilled ultrapure water). 100 µl of the cell suspension was incubated for 30 minutes at room temperature and in the dark by adding 5 µl of Annexin V-FITC (FITC Annexin V, Immunostep, Spain) and propidium iodide (PI) (as in the final concentration of 40 µg/ml, Immunostep, Spain). 1X BB (100 µl) was added into each cell tube and the cells were analyzed using ACEA NovoCyte 3000 Flow cytometer. Data analysis was performed using ACEA NovoExpress software.

### RNA isolation and gene expression analysis by RT-PCR

Total RNA was isolated from HT-29 cells using GeneJET RNA Purification Kit (Thermo Scientific, Cat No: K0731). Concentration and purity of RNA samples were determined using a Take3 Micro-Volume Plate (Epoch, Biotek, Winooski, VT, USA). First strand cDNA was synthesized from 1 μg of RNA using RevertAid First Strand cDNA Synthesis Kit (Fermentas, K1 621). PCR conditions were as follows; 3 min at 94 °C for initial denaturation, 30 s at 94 °C 35 cycles of denaturation, 30 s at 58 °C for annealing, and 45 s at 72 °C for extension. cDNA samples were stored at −20 °C until gene expression analysis. Real-time PCR was performed using ABI 7500 Fast Real Time-PCR and QuantiFast SYBR Green PCR Mix (Qiagen). The primers are shown in Table S1. PCR conditions were as follows; 5 min at 95 °C for initial activation, 10 s at 95 °C for 40 cycles of denaturation and 30 s at 60 °C for combined annealing–extension. All reactions were performed in triplicate and repeated at least 2 times. Cyclophilin A (PPIA) gene was used as an internal control to normalize the target transcripts by the 2^−ΔΔCT^ method^44^.

### Western blot analysis

Total protein extracts from untreated cells or cells treated with EPS at different time intervals (24 h or 48 h) were subjected to Western blot analysis as described by Huang *et al*.^45^. HT-29 cells at a density of 1 × 10^6^ cells were treated with EPSs at a final concentration of 400 µg/ml and incubated for 24 and 48 h. After treatment, the medium was aspirated and the cell culture plate placed on ice washed twice with ice-cold phosphate buffer saline (PBS). After PBS was drained, the cells were lysed by 250 µl of lysis buffer (NP-40 buffer-150 mM NaCl, 50 mM Tris, pH 8.0, and 1% NP-40) containing protein inhibitor cocktail. The cells scraped from the plate gently transferred into the pre-cooled centrifuge tubes. The tubes were kept on ice for 30 min with constant agitation. The lysates were centrifuged at 13,201 × *g* for 20 minutes at 4 °C and the supernatant was stored at 4 °C until use^46,47^. The total protein was determined using the Bradford assay (Sigma-Aldrich). 40 μg of protein lysates denatured in loading buffer at 95°C for 10 min was separated by 4–12% Acrylamide-Bisacrylamide gel and then transferred onto PVDF membranes using iBlot® (ThermoFisher). The membranes were then blocked in blocking solution (Western Breeze, ThermoFisher) at room temperature before incubating with antibodies for one hour. The expression patterns of Bax, Bcl-2, Caspase 3, Caspase 9, Survivin were detected using specific antibodies and β-actin was used as loading control^48^. After washes in Antibody Wash, the membrane was incubated in secondary antibody (anti-rabbit IgG) for 30 min. After second washes, the membrane was incubated in Chromogenic Substrate until the bands develop on the membrane. The molecular weight of the protein bands was determined using BIORAD ImageLab 5.21 program compared to the protein marker (Thermo Scientific PageRuler Prestained Protein Ladder 26616).

### Statistical analysis

All experiments were carried out with three replicates and values were reported as means ± standard deviation (SD), unless otherwise indicated. Statistical analysis was performed using SPSS 16.0 (SPSS Inc., Chicago, IL, USA). Statistical difference was assessed with one-way analysis of variance (ANOVA) followed by Tukey test. For Western blot analysis, t-Test (Excel 2007) was performed. Additionally, p*ost hoc* Dunnett’s test for pair-wise comparison was ran to analyze flow cytometer data. Statistical difference was determined at a P value of 0.05 or less. Monosaccharide composition of EPSs produced by *Lactobacillus* spp. were used to generate a dendrogram by the Ward method of hierarchical clustering (JMP version 12, SAS Institute Inc., Cary, NC).

## Results and Discussion

### Production of EPS by *Lactobacillus* spp. in culture medium and their sugar composition analysis

The bidirectional interaction between host and probiotic bacteria results in health benefits to the host^49^. Several factors in lactobacilli have been shown to impact human health *in vitro* and *in vivo*, including cell surface components and metabolites^49^. EPSs produced by LAB are one of the important component that have a key role in probiotic activity including anti-proliferative effect, immunomodulation, and adhesion^13,17,50^. Here we investigated the production of EPSs by four *Lactobacillus* strains (*L. plantarum* GD2, *L. rhamnosus* E9, *L. brevis* LB63, and *L. delbrueckii* ssp. *bulgaricus* B3) and their compositional analysis. EPSs were freeze-dried and lyophilized. EPS production by various lactobacilli strains differed significantly from each other (p < 0.05). *L. plantarum* GD2, *L. rhamnosus* E9, *L. brevis* LB63, and *L. delbrueckii* ssp. *bulgaricus* B3 yielded 397 ± 4, 298 ± 5, 347 ± 4, and 449 ± 4 mg/l, respectively. *L. delbrueckii* ssp. *bulgaricus* B3 produced the highest amount of EPS among other lactobacilli. *L. delbrueckii* ssp. *bulgaricus* B3 was isolated from yogurt while the others were isolated from feces samples. Mozzi *et al*. reported that most of the HePS are produced by *Lactobacillus* from food origin as seen in our results^51^. Studies on EPS production by *Lactobacillus* to date reported varying amount of EPS. Sungur *et al*. stated that two of the *L. gasseri* strains produced 242 ± 3 mg/l and 255 ± 4 mg/l of lyophilized EPS^15^. In another study, EPS production by *L. crispatus* in different carbon sources ranged from 200 to 400 mg/l^52^. Van Geel-Schutten *et al*. examined the production of EPSs by a total of 182 *Lactobacillus* strains in MRS medium with relatively high sugar concentration^53^. Only 60 of them produced EPS and from those strains only 10% produced EPS more than 100 mg/l which is referred to as the large amount of production. The lactobacilli strains studied here produced in a decent amount of EPS relative to the lactobacilli EPSs studied in the literature^15,52,53^.

EPS produced by LAB exhibit a large variation in their sugar composition^54^. Here, we examined monosaccharide composition of EPSs produced by various *Lactobacillus* spp., using HPLC. Different types of sugar unite were found in the lactobacilli EPSs studied. The EPSs were mainly composed of mannose and glucose ranging between 71–88% and 10–28%, respectively. Additionally, fructose, arabinose, sucrose + maltose, and N-acetylglucosamine were detected at different ratios (Table 1). Detection of maltose and sucrose, disaccharide molecules, might be due to incomplete hydrolysation of EPS. A review on functional properties of EPSs derived from yeast reported that EPSs consisting mannose with more than 50% are classified as biologically active^55^. Anti-cancer activity of EPSs has thought to be related with high amount of mannose in sugar composition^56–58^. Shao *et al*. showed that polysaccharides consisting of glucose and mannose can interact with Toll-like receptors and activate host immunity^56^. Vidhyalakshmi and Vallinachiyar reported that macrophages carry mannose and glucose specific receptors which are important in triggering anti-cancer activities and suppressing cell proliferation in tumor^57^. Similarly, Jin *et al*. studied anti-tumor activity of EPS and concluded that anti-tumor activity of polysaccharides tends to correlate with mannose presence as major monomer in the sugar composition^59^.

**Table 1:** Monomer composition of the EPSs produced by *Lactobacillus* spp.*.

Sugar Composition (%)	Strains			
	GD2_EPS	E9_EPS	LB63_EPS	B3_EPS
Glucose	25.97 ± 0.03	23.63 ± 0.79	27.88 ± 0.03	9.54 ± 0.37
Fructose	ND	ND	ND	1.04 ± 0.45
Mannose	71.03 ± 0.12	74.83 ± 0.81	70.74 ± 0.02	88.25 ± 0.69
Arabinose	2.73 ± 0.15	0.37 ± 0.02	0.67 ± 0.06	ND
Sucrose + maltose	ND	1.05 ± 0.03	0.55 ± 0.02	1.10 ± 0.14
N-acetylglucosamine	0.27 ± 0.01	0.12 ± 0.00	0.17 ± 0.00	0.07 ± 0.00

ND; Not determined.

*Hierarchical clustering of four Lactobacillus spp. strains based on monomer ratio in their EPS sugar composition. The results presented in average.

The presence of different monomers among the EPSs suggest that EPSs of lactobacilli strains studied here are heteropolysacharides as identified mostly in other LAB^54^. Additionally, cluster analysis of sugar composition in EPSs of *Lactobacilli* spp. revealed that the *L. delbrueckii* ssp. *bulgaricus* B3 clustered by itself separated from the other strains (Table 1). Suggesting that this strain tends to have different biological functions compared to the other strains. Based on the variations in the ratio of monomers among various *Lactobacillus*, there is a compositional diversity of EPSs and this diversity likely contributes strain to strain variation in their biological functions including proliferation inhibition and apoptosis induction.

### Structural analysis of EPSs produced by *Lactobacillus* spp

It has been shown that an impressive differences exist in molecular weights and structure of EPSs among LAB^50,60^. To understand better the diversity among *Lactobacillus* spp. and compare the structure of EPSs produced we also performed a molecular weight analysis by SEC and a structural analysis by NMR. SEC results showed that molecular weight of the EPSs ranged from 10^2^ to 10^4^ Da consisting of two fractions except LB63_EPS (Table 2). Interestingly, LB63_EPS has three fractions. Similarly, Tallon *et al*. isolated EPS from *L. plantarum* EP56 that has two fractions with molecular mass of 8.5 × 10^5^ and 4 × 10^4^ Da^61^. However, a study on structure analysis of EPS isolated from another strain of *L. plantarum* (YW32) reported that *L. plantarum* EPS has only one fraction with molecular weight of 1.03 × 10^5^ Da^62^. Hamet *et al*. examined the EPSs of 28 different *Lactobacillus* and showed that the fraction number ranged from one to three with molecular weight distribution being strain dependent^63^. Biofunctinality of EPS has been shown to be affected by molecular weight. Xu *et al*. reported that EPS from *Bifidobacterium animalis* RH has a stronger atioxidant activity due to its low molecular weight^64^. Based on literature, EPS ≤10^4^ Da is considered as low molecular weight^64–66^. It has been reported that low molecular weight polysaccharides can easily pass through the host cell membrane barriers and exhibit biological activity better^30^. On the contrary, in other studies investigating chemical composition and anti-tumor activity of polysaccharides, high molecular weight polysaccharides tend to have more anti-tumor impact than those of low molecular weight^67,68^.

**Table 2:** Molecular weight and linkage type of EPSs produced by *Lactobacillus* spp.

EPSs	Fractions (Dalton, Da)			Linkage Type
	I	II	III	
GD2_EPS	2.4 × 10^3^	2.3 × 10^2^	—	β-(1,3), α-(1,2)
E9_EPS	1.0 × 10^4^	2.7 × 10^2^	—	β-(1,3), α-(1,2)
LB63_EPS	2.5 × 10^3^	9.3 × 10^3^	2.4 × 10^2^	β-(1,3), α-(1,2)
B3_EPS	1.2 × 10^4^	3.5 × 10^2^	—	β-(1,3), α-(1,2)

Furthermore, NMR chemical shifts were determined as described in literature^41,69^ by performing ^1^H-NMR, COSY and NOESY NMR analysis. Generally, the ^1^H NMR spectrum of a polysaccharide can be divided into three main regions: the anomeric region (δ 4.5–5.5), the ring proton region (δ 3.1–4.5) and the alkyl region (δ 1.2–2.3)^70^. In the present study, the chemical shift of the anomeric H1 protons of the EPS was observed at δ 4.8, δ 4.9 and δ 5.2 ppm. ^1^H chemical shifts of the EPS from *Lactobacillus* spp. is shown in Table 3.

**Table 3:** ^1^H chemical shifts of the EPSs from *Lactobacillus* spp.

Proton	Chemical shift (δ) in residue		
	β-D-Mannose	α-D-Mannose	α-D-Glucose
H-1	4.9	5.2	4.8
H-2	4.4	4.1	4.0
H-3	3.9	3.9	3.8
H-4	—	3.8	3.6
H-5	—	3.7	3.4
H-6	—	3.5	3.2

**Figure 1:**
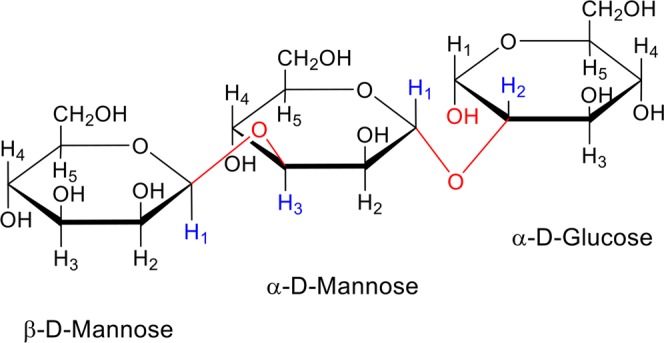
Repeating unit structure of the exopolysaccharides produced by *Lactobacillus* spp. NMR chemical shifts were determined by performing 1H-NMR, COSY and NOESY NMR analysis for the binding stereochemistry of monosaccharide units of EPSs of *Lactobacillus* spp. (*L. plantarum* GD2, *L. rhamnosus* E9, *L. brevis* LB63, and *L. delbrueckii* ssp. *bulgaricus* B3). All of them produced EPS with the same type of sugar linkage which are β-H1-H3 (β-D-Mannose-α-D-Mannose)/α- H1-H2 (α-D-Mannose-α-D-Glucose), designated as β-1,3 (β-D-Mannose-α-D-Mannose) and α-1,2 (α-D-Mannose-α-D-Glucose), respectively.

Similar H–H interactions was observed in all NOESY spectra. The released peaks were observed as a result of interaction between H1 proton of α-D-mannose (5.2 ppm) and H2 proton of α-D-glucose (4.0 ppm protons). Furthermore, the H3 proton of α-D-mannose and H1 proton of β-D-mannose were found to be in interaction. These results helped us to understand the sequencing and binding stereochemistry of monosaccharide units of EPSs of *Lactobacillus* spp. The results showed that all of the lactobacilli studied here produced EPS with the same type of sugar linkage which are β-H1-H3 (β-D-Mannose-α-D-Mannose)/α-H1-H2 (α-D-Mannose-α-D-Glucose), designated as β-1,3 (β-D-Mannose-α-D-Mannose) and α-1,2 (α-D-Mannose-α-D-Glucose), respectively. The primary structure of EPSs is shown in Fig. 1.

Two types of linkage were found among *Lactobacillus* spp. Main sugar linkage types present in the EPS fractions isolated from *Lactobacillus* spp. are β-(1,3) and α-(1,2) (Table 2). Compared to β-linkages, α-linkages result in more flexible polymers^22^. It has been shown that molecular properties including type of linkages of the polysaccharides strongly impact the interactions with proteins^71^. Biological activities such as anti-tumor and apoptosis inducing activity by polysaccharides are strongly associated with their structures. It has been reported that anti-tumor polysaccharides mainly show β-1,3-linkages and polysaccharides containing mainly β-1,6- linkages have less anti-tumor activities^71,72^. Another study mainly focused on the structure of β-glucan demonstrated that (1,3)- β-glucan with the (1,6)- β-glucan branches increased immuno competent cell activity and have an important role in anti-tumor activity of the polysaccharides^67^. However, anti-tumor activity of EPS with different linkage have also been reported such as EPS from *L.plantarum* with β-D-(1–4), β-D-(1–6)-linked glucose residues^73^. As a result, similar linkage structure was determined in all EPSs used in this study. Variations in the ratio of the monomers and molecular weight among *Lactobacillus* spp. strains suggest that the compositional diversity of EPSs isolated from *Lactobacillus* spp. likely contributes strain to strain variation in their ability to inhibit proliferation and induce apoptosis. To better understand the contribution of this variation, we further analyzed the impact of EPSs of *Lactobacillus* spp. on colon cancer cells, HT-29.

### Anti-proliferative impact of EPSs produced from *Lactobacillus* spp. strains on HT 29 cells

**Figure 2:**
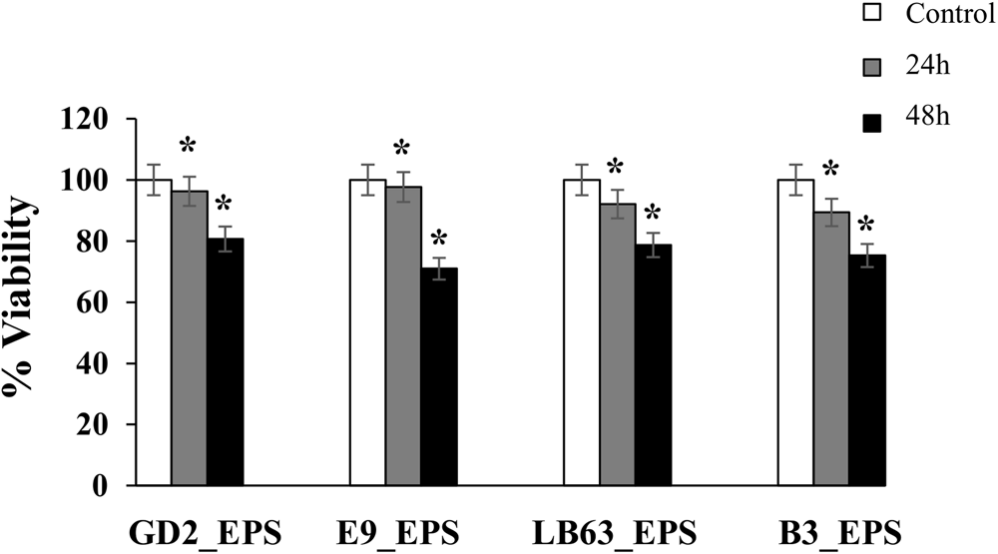
Anti-proliferative effect of the EPSs produced by *Lactobacillus* spp. against HT-29 colon cancer cells at two time points. HT-29 cells seeded at a density of 1 × 10^4^ cells/well were treated with EPSs of *Lactobacillus* spp. (*L. plantarum* GD2, *L. rhamnosus* E9, *L. brevis* LB63, and *L. delbrueckii* ssp. *bulgaricus* B3) at a final concentration of 400 µg/ml for 24 h or 48 h and the anti-cytotoxicity effect of the EPSs was evaluated by WST-1 assay. *p < 0.05, significant difference from the control (n:3 for each bar).

Studies have suggested that LAB products including EPS have anti-tumor activity^21,41,74^. Researches on the impact of EPS on therapeutic functions including anti-tumor activity and immunomodulation brings new expectations to biomedical fields^18,73,75^. Here, we examined the cytotoxic effect of EPSs from *Lactobacillus* spp. on HT-29 cells at two time points, 24 h and 48 h by WST-1 assay. EPSs showed an anti-proliferative effect on HT-29 cells in a time dependent manner and differed significantly (p < 0.05) from the control (Fig. 2). The cell death in the cells exposed to GD2_EPS, E9_EPS, LB63_EPS, and B3_EPS was higher at 48 h time point than at 24 h time point, with 80.7 ± 1.8%, 71 ± 1.6%, 78.7 ± 1.9%, and 75.3 ± 1.7% cell viability, respectively. Wang *et al*. tested the inhibitory effect of EPS from *L. plantarum* strain against HT-29 cells for two time intervals, 24 h and 72 h. While they barely saw an impact at 24 h time point, the strongest anti-proliferative impact was seen after 72 h^62^. However, it is not known that if the cell death happened due to necrosis or apoptosis. As mentioned previously, type of the linkage in lactobacilli EPS may correlate with the anti-tumor activity. β-1,3-linkage has been shown to have a better anti-tumor activity^71,72^. All of the strains we studied here contain β-(1,3), α-(1,2) as the main linkage. The capability of the *Lactobacillus* to inhibit proliferation in HT-29 cells are likely to be related with their structure. In addition to the chain linkage, anti-proliferative effect of polysaccharides has been demonstrated to be related to their chemical characteristics including molecular weight and molecular composition^28^. Here in this study we showed that *Lactobacillus* varied in molecular weight and sugar composition of their EPSs. This variation might have impact on their different anti-proliferative effect. Overall, these results suggest that all of the EPSs from the lactobacilli strains studied here are capable of inhibiting proliferation of HT-29 cells in a time dependent manner.

### Impact of EPS produced from *Lactobacillus* spp. strains on apoptosis in HT-29 cells

**Figure 3:**
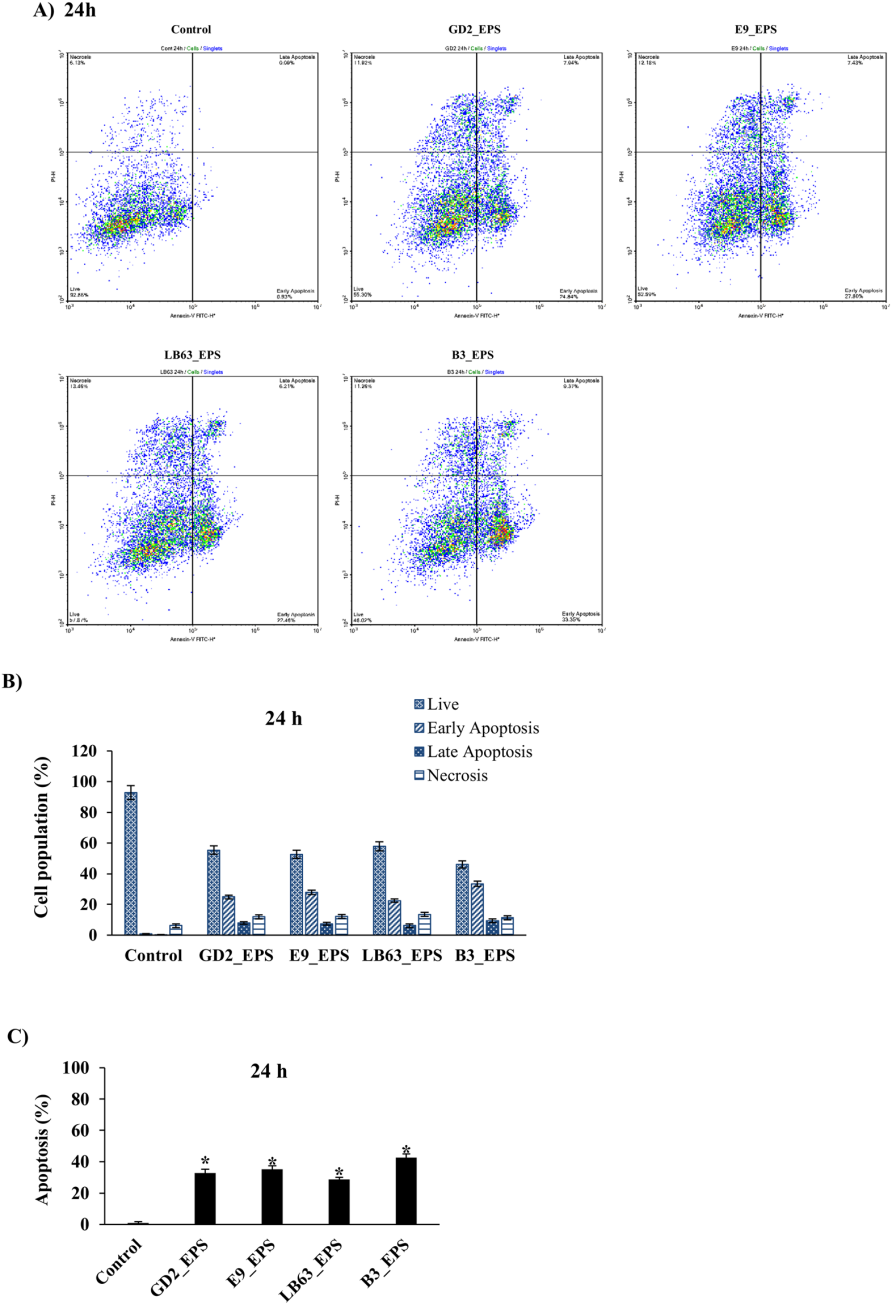
Flow cytometric analysis of the impact of *Lactobacillus* spp. EPSs on apoptosis in HT-29 cells at 24 h time point. HT-29 cells seeded at a density of 1 × 10^4^ cells/well were treated with EPSs of *Lactobacillus* spp. (*L. plantarum* GD2, *L. rhamnosus* E9, *L. brevis* LB63, and *L. delbrueckii* ssp. *bulgaricus* B3) at a final concentration of 400 µg/ml for 24 h and then stained with Annexin V-FITC and PI. Fluorescence intensities were detected by flow cytometry to determine the effect of the EPSs on earlier apoptosis, late apoptosis and necrosis. (**A**) Distribution of viable, early apoptotic, late apoptotic and necrotic cells analyzed by flow cytometry. (**B**) Percentage of cells in viable, early apoptotic, late apoptotic and necrotic stages. (**C**) Percentage of apoptosis in HT-29 cells exposed to the lactobacilli EPSs for 24 h. *p < 0.05, significant difference from the control.

**Figure 4:**
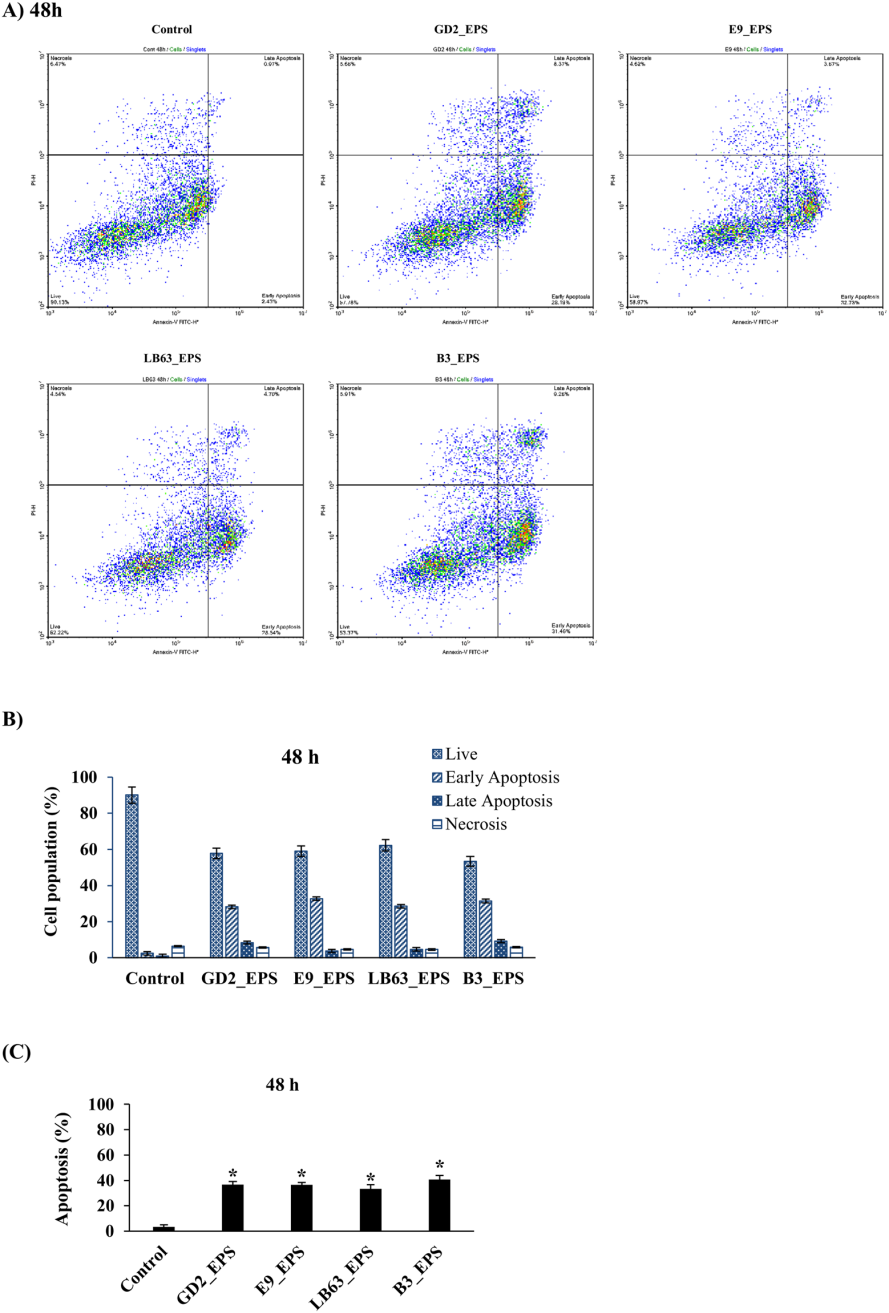
Flow cytometric analysis of the impact of *Lactobacillus* spp. EPSs on apoptosis in HT-29 cells at 48 h time point. HT-29 cells seeded at a density of 1 × 10^4^ cells/well were treated with EPSs of *Lactobacillus* spp. (*L. plantarum* GD2, *L. rhamnosus* E9, *L. brevis* LB63, and *L. delbrueckii* ssp. *bulgaricus* B3) at a final concentration of 400 µg/ml for 48 h and then stained with Annexin V-FITC and PI. Fluorescence intensities were detected by flow cytometry to determine the effect of the EPSs on earlier apoptosis, late apoptosis and necrosis. (**A**) Distribution of viable, early apoptotic, late apoptotic and necrotic cells analyzed by flow cytometry. (**B**) Percentage of cells in viable, early apoptotic, late apoptotic and necrotic stages. (**C**) Percentage of apoptosis in HT-29 cells exposed to the lactobacilli EPSs for 48 h. *p < 0.05, significant difference from the control.

Apoptosis is a programmed cell death and is crucial in development and tissue homeostasis^76^. Induction of apoptosis could be used in control of proliferation of cancer cells. Most anti-cancer drugs in use affect via the induction of apoptosis^77,78^. To further analyze the anti-proliferative effect by EPSs of *Lactobacillus* spp., we treated the HT-29 cells with EPSs at two incubation time points, 24 h or 48 h, and performed a flow cytometric analysis. EPSs of *Lactobacillus* spp. induced apoptosis in HT-29 cells at both time points with an apoptosis percentage ranging from 28 to 43 at 24 h and 33 to 41 at 48 h. (Figs 3B, 4B). EPSs from *L. delbrueckii* ssp. *bulgaricus* B3 showed the highest apoptosis percentage (42.9 ± 2.4% and 40.6 ± 3.3, respectively) at both time points. Distribution of viable, early apoptotic, late apoptotic and necrotic cells showed that EPSs of *Lactobacillus* spp. induced both early and late apoptosis in HT-29 cells with the early apoptosis being higher (Figs 3C, 4C). Our results suggest that anti-proliferative impact of *Lactobacillus* studied here could be due to their capability to induce apoptosis (Figs 2–4). This is important in developing a new anti-cancer drug with good efficacy leading the cancer cells to apoptosis.

### Genes and proteins involved in apoptotic pathway induced by EPSs of *Lactobacillus* spp

To further understand the underlying mechanism of EPS-induced apoptosis, we next investigated changes in gene expression of apoptosis markers in HT-29 cells treated with EPSs of *Lactobacillus* spp. We targeted five genes associated with the apoptotic pathway, which are Bax, Bcl-2, Caspase 3, Caspase 9, and Survivin and measured both relative gene expression and protein expression.

**Figure 5:**
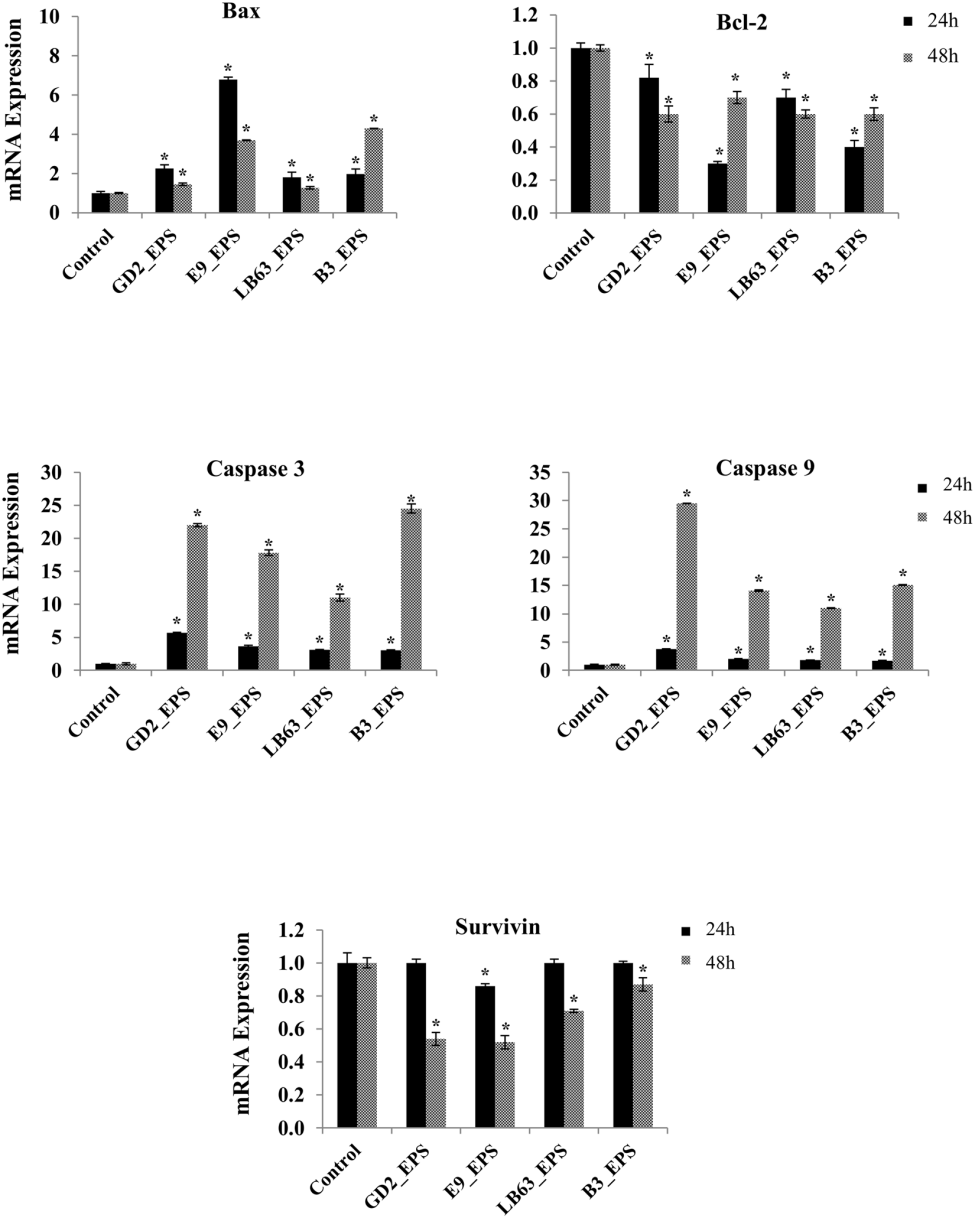
Fold change in mRNA expression of target genes of the HT-29 cells in the control group and the group treated with EPSs from *Lactobacillus* spp. (*L. plantarum* GD2, *L. rhamnosus* E9, *L. brevis* LB63, and *L. delbrueckii* ssp. *bulgaricus* B3) at a final concentration of 400 µg/ml for 24 h (black bar) or 48 h (gray bar). *p < 0.05, significant difference from the control (n: 3).

**Figure 6:**
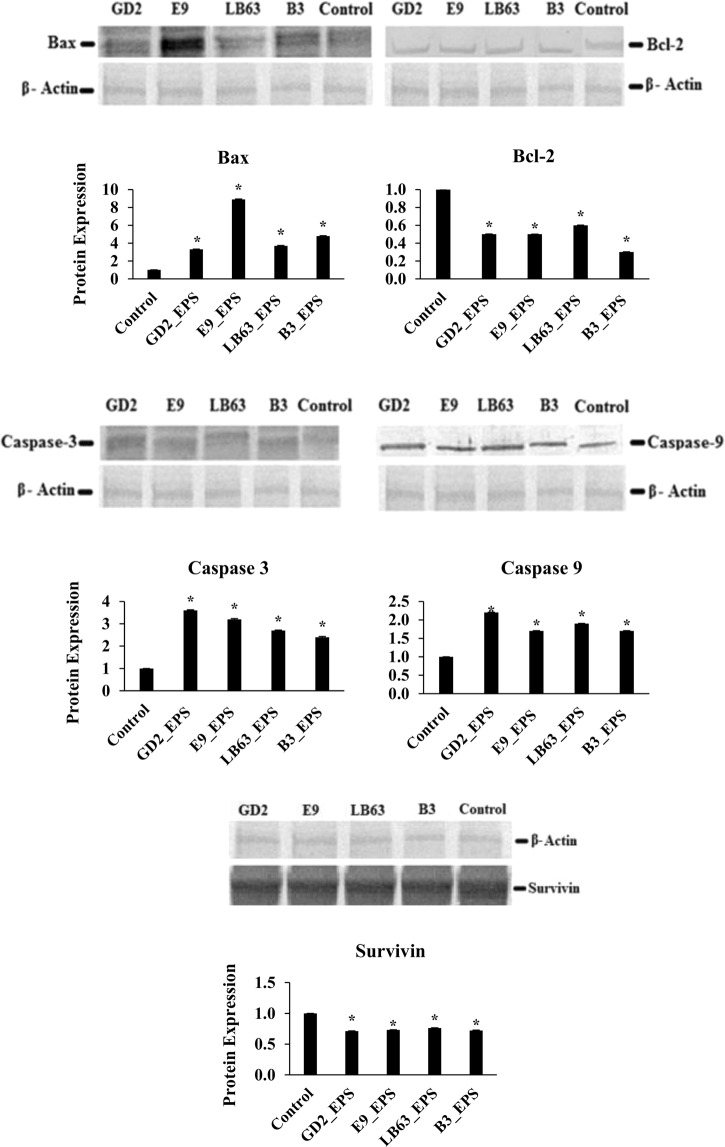
Protein expression of target genes of the HT-29 cells in the control group and the group treated with EPSs from *Lactobacillus* spp. (*L. plantarum* GD2, *L. rhamnosus* E9, *L. brevis* LB63, and *L. delbrueckii* ssp. *bulgaricus* B3) at a final concentration of 400 µg/ml for 24 h. *p < 0.05, significant difference from the control (n:3).

Bcl-2 is a large family of proteins that regulate cell death and cell survival. Bcl-2 family proteins take an important role in mitochondria-mediated apoptotic pathway and control the integrity of the mitochondrial outer membrane^79^. In this study, two of Bcl-2 family proteins were examined, Bcl-2 which is an anti-apoptotic protein and Bax which is a pro-apoptotic protein. Effect of EPSs from *Lactobacillus* spp. on both anti-apoptotic and pro-apoptotic Bcl-2 family proteins were analyzed. The gene expression results showed that *Lactobacillus* spp. significantly increased the Bax gene expression at two time points, relative to the control, non-treated HT-29 cells (Fig. 5). However; a significant decrease in expression of Bcl-2 gene was observed in HT-29 cells treated with EPSs of *Lactobacillus* spp. for 24 h or 48 h, relative to the control (Fig. 5). The induction of Bax gene was higher at 24 h time point compared to 48 h time point. The highest increase of Bax gene expression at 24 h time point was observed in the HT-29 cells treated with EPS of *L. rhamnosus* E9, which was 6.79 ± 0.12 -fold change relative to the control (Fig. 5). The highest increase of Bax gene expression at 48 h time point was observed in the HT-29 cells treated with EPS of *L. delbrueckii ssp. bulgaricus* B3, which was 4.3 ± 0.01-fold change relative to the control (Fig. 5). Once apoptosis is induced by any agent, Bax proteins reach the mitochondrial outer membrane from cytoplasm. At the same time, anti-apoptotic Bcl-2 family proteins such as Bcl-2 binds Bax to prevent their transfer to the mitochondrial membrane. Therefore, in a cell underwent apoptosis, an increase in Bax gene and a decrease in Bcl-2 gene is expected. Bax/Bcl-2 ratio, hence, could be used to determine the fate of the cells in the apoptotic system^80^. Protein expression data confirmed these results (Figs 6 and S1).

Most of the molecules taking role in cell death are controlled by caspases. Apoptotic caspases can be grouped in two categories; caspases that initiate apoptosis are Caspase 2, 8, 9, and 10; and effector caspases with Caspase 3, 6, and 7^81^. We examine the Caspase 3 and 9 gene expression in HT-29 cells to evaluate the impact of EPSs of *Lactobacillus* spp. on expression of genes encoding caspases. Of the caspases examined, all of the *Lactobacillus* spp. EPSs resulted in a significant increase in gene expression at both 24 h (Fig. 5) and 48 h time point (Fig. 5). Levels of Caspase 3 and 9 gene expression at 24 h time point were the highest in the HT-29 cells treated with EPS of *L. plantarum* GD2, which were 5.68 ± 0.07 and 3.77 ± 0.09 -fold change relative to the control, respectively. At 48 h time point, both *L. plantarum* GD2 and *L. delbrueckii ssp. bulgaricus* B3 EPSs resulted in a high increase in Caspase 3 with 22 ± 0.26 and 24.5 ± 0.69 - fold change, respectively. The highest Caspase 9 gene expression was in HT-29 cells treated with EPS of *L. plantarum* GD2 with 29.5 ± 0.05 -fold change relative to the control (Fig. 5). Protein expression results also confirmed the gene expression data (Figs 6 and S1).

Studies have shown that polysaccharides induce apoptosis in cancer cells in a time dependent manner^32,45^. In this study, administration of lactobacilli EPSs to HT-29 cells resulted in a time dependent induction in expression of caspases with a higher impact at 48 h compared to the impact at 24 h. The results indicate that EPSs induce apoptosis by caspase activation. Mitochondrial depolarization was dependent on caspase activation, suggesting a positive amplification loop for mitochondrial dysfunction. Loss of mitochondrial membrane potential would lead to release of cytochrome *C* and activation of Bax and Caspase 3/9^82^.

Survivin is a member of apoptosis inhibitor family^76^ and inhibits Caspase 3 and 9. The expression of Survivin gene was suppressed by the EPS of *L. rhamnosus* E9 (only 0.14 ± 0.01 fold) at 24 h (Fig. 5). However, all of the EPSs resulted in a significant suppression (p < 0.05) in the gene expression of Survivin at 48 h (Fig. 5). This suggest that suppression of Survivin just started at 24 h and reached a high suppression level at 48 h. However, the protein suppression of Survivin has started earlier than the suppression of the gene expression did (Figs 6 and S1). Similarly, Stolfi et al. showed that reduction of Survivin protein was seen as early as 8 h following the application of an apoptosis inducing agent whereas the inhibition of Survivin gene expression occurred at later time points (i.e., 32 hours)^83^ suggesting that a posttranscriptional control of Survivin could be involved in this process. EPS from *Aphanothece halaphytica* has been shown to induce apoptosis in cancer cells by modulating p53-survivin pathway and target unfolded Protein Response Regulator Grp78^84^. The EPS of *A*. *halaphytica* induce the expression of CHOP and suppress the expression of Survivin, which leads p53-survivin pathway and cause apoptosis by activating Caspase 3.

The gene and protein expression results demonstrated that the ability to induce apoptosis by EPSs of *Lactobacillus* spp. associated with an upregulation of Bax, Caspase 3, and 9 and a downregulation of Bcl-2 and Survivin. *In vitro* studies have suggested that colon cancer could be inhibited by activation of Caspase 3 and 9 and inhibition of Bcl-2^85–87^. In another study, EPS isolated from *A. halophytica* induce apoptosis similar to our study. They reported that endoplasmic reticulum (ER) signaling pathway leads apoptosis. Based on their explanation for the molecular mechanism, ER targets Grp78 regulating cell respond UPR and suppresses Survivin and Bcl-2 expression while induces Caspase 3 expression, as a results, leads the cells to apoptosis^84^. Our results suggest that EPSs released by *Lactobacillus* spp. studied here inhibit proliferation via apoptosis in HT-29 cells. It has been reported that biofunctional activities of polysaccharides including anti-tumor and apoptosis inducing activity are strongly associated with their molecular weight, monomer composition, structure, and linkage type^67,72,73,88^. The capability of EPSs from *Lactobacillus* spp. to induce apoptosis at a high level, relative to the EPSs inducing apoptosis in the literature, might be associated with the main monomer in their structure, mannose, and their linkage type which are β-(1,3), α-(1,2)^75,88–90^. Glucose and mannose are known to have highly specific receptors on macrophages, which is important in tumor immunology. The ability of all EPSs studied here to induce apoptosis in colon cancer cells could be related to their sugar composition having mannose and glucose as major component. B3_EPS, which has the highest amount of mannose in sugar composition, showed the highest apoptosis induction on HT-29 cells. Suggesting that there might be a relationship exists between the ability of an EPS to induce apoptosis and its mannose composition. Additionally, B3_EPS has the lowest amount of glucose relative to the other strains. Having low amount of glucose with a high mannose content might have a role in the ability of B3_EPS to induce apoptosis strongly.

## Conclusion

EPSs produced by LAB are one of the important component that have a key role in probiotic activity including anti-tumor effect, immunomodulation, and adhesion^13,17,50^. In this study we evaluated EPSs of four previously described *Lactobacillus* spp. isolated from healthy infant feces (GD2_EPS, E9_EPS, and LB63_EPS) and yogurt (B3_EPS) for their health effect on colon cancer cells (HT-29) and for their physicochemical properties^34,35^. We demonstrated that a compositional and structural diversity exists within *Lactobacillus* spp., and this diversity likely contributes to variation in the ability to inhibit proliferation and induce apoptosis. Relative proportions of the individual sugars among *Lactobacillus* spp. are different and mannose of which portion size impact biological activities^55^, is the major sugar component in EPSs. All of the EPSs contain β-1,3-linkage which has been found in anti-tumor polysaccharides^71,72^. The results showed that EPSs of *Lactobacillus* spp. inhibit proliferation in colon cancer cells via apoptosis. The level of their capability to induce apoptosis was time dependent. While B3_EPS and GD2_EPS have better impact on inducing Caspases, E9_EPS and B3_EPS showed better impact on Bax and Survivin modulation. B3_EPS showed the highest apoptosis induction on HT-29 cells and has the highest amount of mannose in sugar composition with a quite low amount of glucose. There might be a relationship exists between the ability of an EPS to induce apoptosis and its high mannose and low glucose composition. Collectively these findings are important for further evaluating *Lactobacillus* spp. EPSs for cancer therapy depending on EPS structure and monomer composition. Particularly mannose ratio in EPS composition should be taken into consideration when designing anti-cancer agents. Further research is required to elucidate the mechanism of action of mannose and glucose on anti-cancer functionality.

## Supplementary information

Table S1, Figure S1, and Figure S2

## Data Availability

All data generated or analysed during this study are included in this published article (and its Supplementary Information files).
