# Supplementary material for: The relationship between the structural characteristics of lactobacilli-EPS and its ability to induce apoptosis in colon cancer cells in vitro
Source: Sci Rep. 2019 Jun 4;9:8268. doi: 10.1038/s41598-019-44753-8 (PMC6547643; doi:10.1038/s41598-019-44753-8)
Supplement: Supplementary file 1 — Table S1, Figure S1, and Figure S2 [file 41598_2019_44753_MOESM1_ESM.pdf]

The relationship between the structural characteristics of lactobacilli-EPS and its ability to induce apoptosis in colon cancer cells *in vitro*

Ummugulsum Tukenmez[a], Busra Aktas[b*], Belma Aslim[a], Serkan Yavuz[c]

[a] Faculty of Science, Department of Biology, Gazi University, Ankara, Turkey
[b] Faculty of Arts and Science, Department of Molecular Biology and Genetics, Burdur Mehmet Akif Ersoy University, Burdur, Turkey
[c] Faculty of Science, Department of Chemistry, Gazi University, Ankara, Turkey
[*]Corresponding author. Email: aktas@uwalumni.com

Table S1. qPCR Primer sequences and amplicon sizes of target genes

**Genes**	**Forward Primer (5'-3')**	**Reverse Primer (5'-3')**	**Amplicon Size (Bp)**
PPIA	5'- GGTCCCAAAGACAGCAGAAA-3'	5'- GTCACCACCCTGACACATAAA-3'	115
Bax	5'- TTCTGACGGCAACTTCAACT -3'	5'- CAGCCCATGATGGTTCTGAT -3'	117
Bcl-2	QuantiTect QT00025011 (Qiagen)	QuantiTect QT00025011 (Qiagen)	117
Casp3	5'-GCGAATCAATGGACTCTGGA -3'	5'-TTTGCTGCATCGACATCTGTA -3'	148
Casp9	QuantiTect QT00036267 (Qiagen)	QuantiTect QT00036267 (Qiagen)	130
Survivin	5'-CCACCGCATCTCTACATTCA-3'	5'-CCAAGTCTGGCTCGTTCTC-3'	116

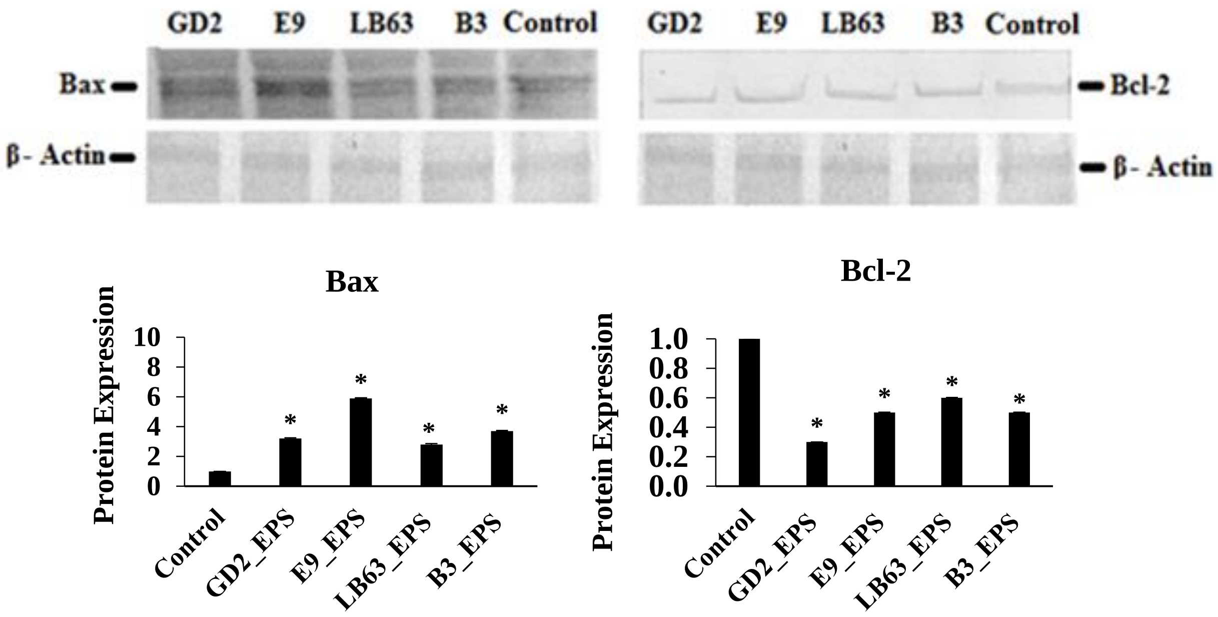
GD2 E9 LB63 B3 Control
GD2 E9 LB63 B3 Control
Bax
β- Actin
Bcl-2
β- Actin
Bax
Protein Expression
10
8
6
4
2
0
Control
GD2_EPS
E9_EPS
LB63_EPS
B3_EPS
Bcl-2
Protein Expression
1.0
0.8
0.6
0.4
0.2
0.0
Control
GD2_EPS
E9_EPS
LB63_EPS
B3_EPS

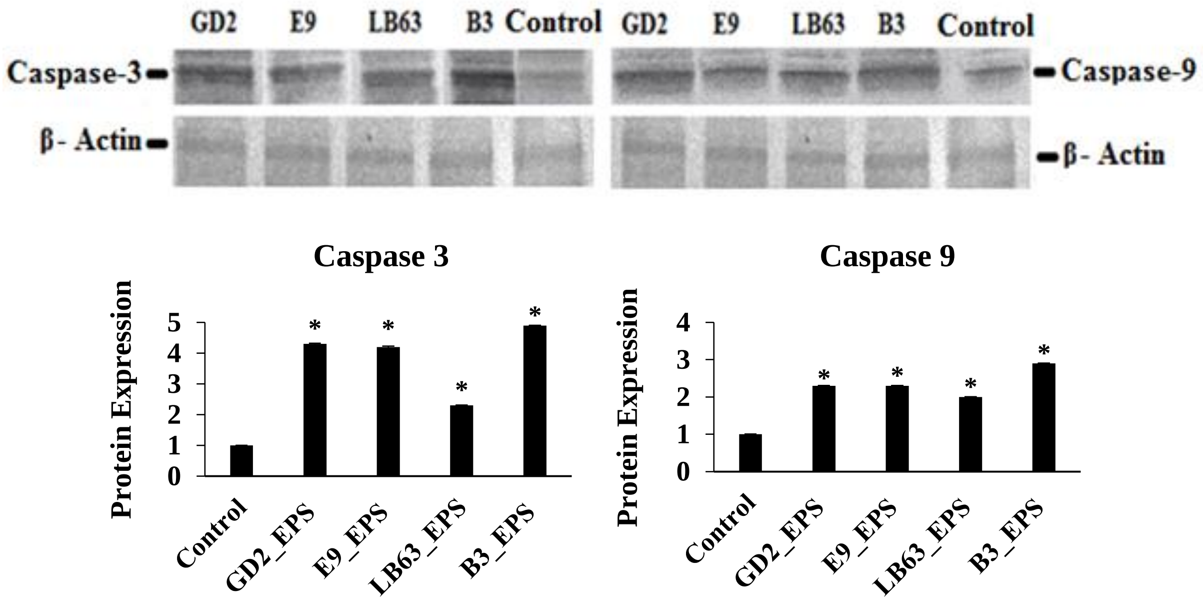
GD2 E9 LB63 B3 Control
GD2 E9 LB63 B3 Control
Caspase-3
β- Actin
Caspase-9
β- Actin
Caspase 3
Protein Expression
5
4
3
2
1
0
Control
GD2_EPS
E9_EPS
LB63_EPS
B3_EPS
Caspase 9
Protein Expression
4
3
2
1
0
Control
GD2_EPS
E9_EPS
LB63_EPS
B3_EPS

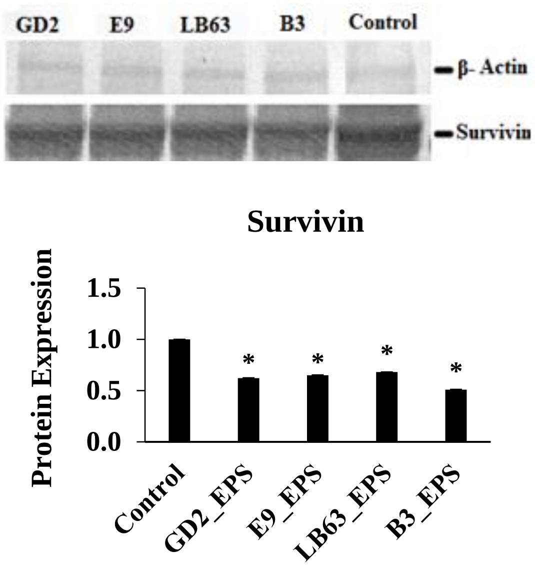

Fig S1. Expression of target proteins of the HT-29 cells after 48h administration of EPSs from *Lactobacillus* spp. * $p<0.05$, significant difference from the control (n: 3)

The gel images have not been cropped from different gels. Since every single gel was used for more samples than studied in this manuscript, we only removed the samples that are not used in this manuscript (which will be used in another manuscript under preparation) from the gel as seen in the raw data example for western images_bax protein.

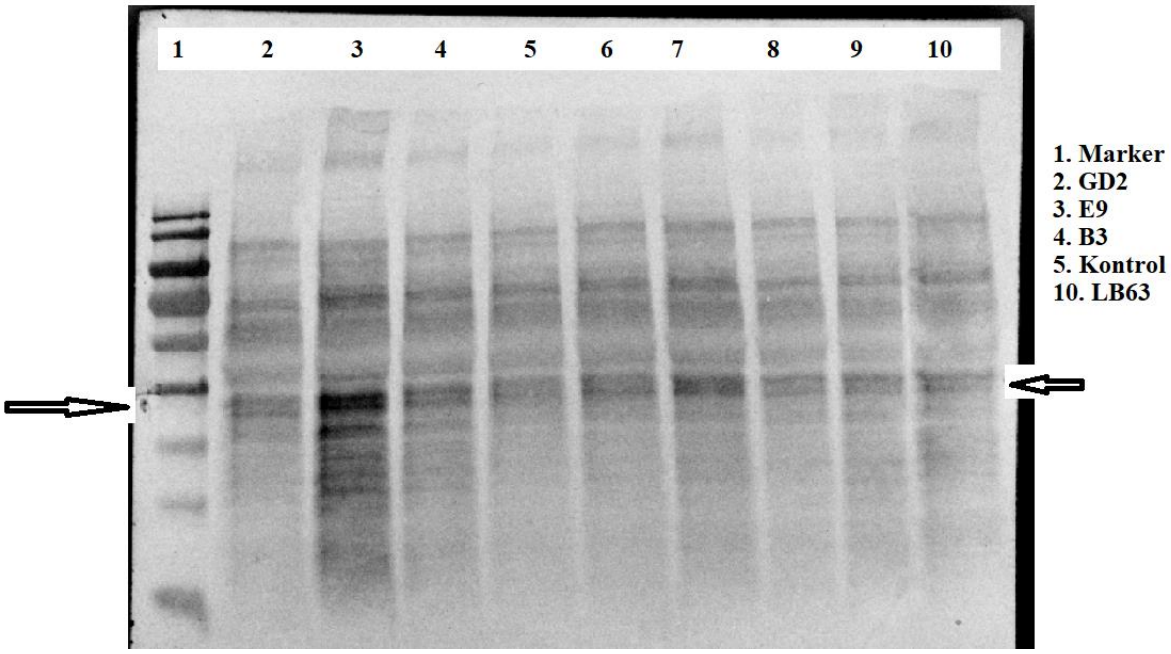

Fig S2. Expression of bax protein of the HT-29 cells after 24h administration of EPSs from *Lactobacillus* spp.
